# Sex differences in HDL ApoC-III in American Indian youth

**DOI:** 10.1186/2042-6410-3-18

**Published:** 2012-08-16

**Authors:** Piers R Blackett, Sohail Khan, Wenyu Wang, Petar Alaupovic, Elisa T Lee

**Affiliations:** 1 Department of Pediatrics, University of Oklahoma Health Sciences Center, OU Children’s Physician’s Bldg, 1200 N Phillips Ave, Oklahoma City, OK, 73104, USA; 2 Cherokee Nation Capitol, P.O. box 948, Tahlequah, OK, 74465, USA; 3 Center for American Indian Health Research, College of Public Health, University of Oklahoma Health Sciences Center, Oklahoma City, OK, 73104, USA; 4 Oklahoma Medical Research Foundation, 825 NE 13th St., Oklahoma City, OK, 73104, USA; 5 Center for American Indian Health Research, College of Public Health, University of Oklahoma Health Sciences Center, Oklahoma City, OK, 73104, USA

**Keywords:** Gender, HDL, ApoC-III, Children, Adolescents

## Abstract

**Background:**

Since American Indians are predisposed to type 2 diabetes (DM2) and associated cardiovascular risk, Cherokee boys and girls (n = 917) were studied to determine whether BMI Z (body mass index Z score) is associated with the apoC-III (apolipoprotein C-III) content of HDL (high density lipoprotein), a previously reported predictor of DM2.

**Methods:**

An *ad hoc* cross-sectional analysis was conducted on a previously studied cohort. Participants were grouped by gender-specific age groups (5 to 9, 10 to 14 and 15 to 19 years). ApoA-I (apolipoprotein A-I) and HDL apoC-III were assayed by electroimmunoassay. ApoC-III was measured in whole plasma, and in HDL to determine the molar proportion to apoA-I. General linear models were used to assess association.

**Results:**

The HDL apoC-III to apoA-I molar ratio increased by BMI Z quartile in girls aged 10–14 years (p < 0.05 for linear trend, p < 0.05 for difference in BMI Z quartile IV vs. I to III) and aged 15–19 years (p < 0.05 for trend). In boys the increase by BMI Z occurred only at ages 15–19 years (p < 0.01 for trend and for quartile difference).

**Conclusions:**

ApoC-III showed an obesity-related increase relative to apoA-I during adolescence beginning in girls aged 10 to 14 years and in boys aged 15 to 19 years. The earlier changes in girls may alter HDL’s protective properties on the β-cell and contribute to their increased risk for DM2.

## Background

It is known that cardiovascular risk factors precede the onset of DM2 [1], and it has been proposed that CVD and DM2 share common antecedents [2]. Furthermore the presence of risk factors leading to the early onset of DM2 has been increasingly observed in adolescents [3], especially in American Indian youth [4] stressing the importance of identifying risk markers for enhancing early detection and prevention in this population.

The hypothesis that a low HDL-C (HDL cholesterol) plays a role in the onset of DM2 is supported by findings in the Prospective Cardiovascular Münster (PROCAM) study on middle-aged men showing that low HDL-C was an independent risk factor for DM2 and was interactive with BMI [5]. However there is limited information on the apolipoprotein content of HDL in relation to obesity and possible diabetes risk. This *ad hoc* cross-sectional analysis of cardiovascular and diabetes risk factors in the Cherokee Diabetes Study included HDL apoC-III, which is of potential significance since it has recently been shown to predict DM2 in a Turkish population in whom the highest tertile conferred a 2.5-fold risk ratio for one standard deviation increment with a greater effect in women [6]. The prediction was independent of obesity and greater than that due to waist circumference. Based on their conclusion and hypothesis, we perceived a need for replicating this important finding. However, in our cross-sectional observations on the Cherokee children and adolescents we were limited to investigating association of HDL apoC-III with the pre-diabetes phenotype manifesting as obesity associated with insulin resistance progressing to a decline in insulin levels.

It is concerning that an increased prevalence of DM2 has been observed in females in five North American populations [4]. This is attributed in part to β-cell deterioration during adolescence, a critical period of development, thus increasing risk for DM2 [7]. Since the markedly increased female incidence of DM2 is unexplained, we evaluated gender differences in HDL apoC-III in American Indian children and adolescents, to determine whether it is differently associated with obesity between sexes.

## Methods

With collaboration of the Cherokee Nation of Oklahoma, a total of 2,205 participants aged 5–40 years volunteered to participate in the Cherokee Diabetes Study. Eleven subjects with DM2 as defined by a fasting glucose greater than 126 mg/dl were excluded [8]. 975 non-diabetic male and female subjects aged 5–19 years were studied allowing sufficient power for subgrouping and making it unlikely that rare genetic forms of obesity would have an effect.

Informed consent was obtained from each subject or his/her legal guardian, following approval of the Institutional Review Boards of the University of Oklahoma Health Sciences Center and the Cherokee Nation. Standard methods for conduction of the study, reporting and data deposition were adopted according to the study operations manual.

### Body mass index (BMI)

Weight and height were determined using accurate standardized methods according to the operations manual. BMI was calculated from the weight in kilograms divided by the height in meter squared. Since there is a known close to linear increase in BMI during late childhood and adolescence, the age adjusted BMI Z score was computed by formula considering variation from the median.

### Lipids and apolipoproteins

Lipid assays included triglycerides, cholesterol, and HDL-C. An Abbott VP-Super System automatic analyzer and commercial reagents were used to determine levels of cholesterol (Boehringer, Mannheim, Federal German Republic) and triglyceride (Miles Inc., Tarytown, NJ) by enzymatic methodology. HDL-C was measured following the heparin-manganese precipitation procedure of the Lipid Research Clinics program and LDL-C (low density lipoprotein cholesterol) was calculated by the Friedewald formula. ApoA-I [9], and apoC-III [10] were determined by electroimmunoassays.

### Glucose and insulin

Fasting insulin levels were determined in the National Institutes of Health core laboratory at the Endocrinology Department, University of Chicago, Chicago, IL. Insulin was measured in serum samples using an overnight competitive double antibody radio-immunoassay [11] using a modification of the procedure and glucose by an automated method using glucose oxidase (Alfa Wassermann, Inc., West Caldwell, NJ).

### Statistical analyses

Data were grouped by sex and categorized by age from 5–9 years, 10–14 years and 15 to 19 years . General linear models were used to assess the association of BMI Z with HDL apoC-III.

## Results

### Lipids

Triglycerides were highest (p < 0.01) for participants with BMI Z scores in the 4^th^ quartile in both sexes, and HDL-C values was lowest in 4^th^ quartile (p < 0.01) for both sexes with the exception of boys aged 15 to 19 years. Cholesterol was not different (Table 1).

**Table 1:** Cholesterol, triglyceride and HDL-C by sex, age group and BMI Z quartiles I-III and IV

	**Sex**	**Age**	**BMI Z quartiles**				
		**(years)**	**I-III**	**n**	**IV**	**n**	**p**
Total	Females	5-9	147.4 + 23.5	95	148.9 + 25.3	32	0.7586
cholesterol		10-14	138.6 + 23.3	138	147.5 + 32.9	46	0.0919
(mg/dl)		15-19	142.1 + 28.9	120	150.5 + 31.4	41	0.1336
	Males	5-9	151.4 + 27.5	91	152.2 + 28.2	31	0.8967
		10-14	142.1 + 28.7	135	148.3 + 31.3	45	0.2425
		15-19	144.3 + 28.9	97	156.8 + 31.9	32	0.0513
Triglycerides	Females	5-9	58.1 + 37.4	95	85.9 + 60.0	32	
(mg/dl)		10-14	70.3 + 38.0	138	108.7 + 62.8	46	
		15-19	69.3 + 37.8	120	92.8 + 37.6	41	
	Males	5-9	47.8 + 20.6	91	78.2 + 41.1	31	
		10-14	62.1 + 30.0	135	93.2 + 43.9	45	
		15-19	76.2 + 35.6	97	122.3 + 78.6	32	
Log triglycerides	Females	5-9	3.91 + 0.54	95	4.28 + 0.59	32	0.0026
		10-14	4.13 + 0.49	138	4.54 + 0.56	46	0.0001
		15-19	4.12 + 0.48	120	4.46 + 0.37	41	0.0001
	Males	5-9	3.76 + 0.48	91	4.24 + 0.49	31	0.0001
		10-14	4.02 + 0.45	135	4.42 + 0.51	45	0.0001
		15-19	4.23 + 0.47	97	4.63 + 0.58	32	0.0007
HDL-C							
(mg/dl)	Females	5-9	47.0 + 9.4	95	39.5 + 9.2	32	0.0002
		10-14	43.2 + 9.4	138	38.1 + 7.5	46	0.0003
		15-19	44.7 + 8.6	120	39.1 + 9.6	41	0.0018
	Males	5-9	51.0 + 11.4	91	39.3 + 8.3	31	0.0001
		10-14	44.8 + 9.9	135	37.5 + 6.6	45	0.0001
		15-19	40.2 + 8.7	97	36.7 + 8.8	32	0.0508

The p values are derived from testing the difference in means of those with BMI Z quartiles I-III vs. IV.

### ApoA-I and ApoC-III

**Figure 1:**
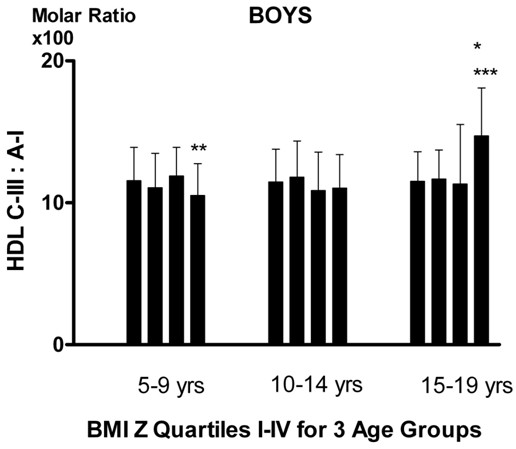
HDL ApoC-III: ApoA-I molar ratio (x100) by BMI Z quartiles in boys.* p for trend < 0.05, ** p < 0.05 for difference between quartiles I-III and IV, *** p < 0.01 for difference between quartiles I-III and IV.

**Figure 2:**
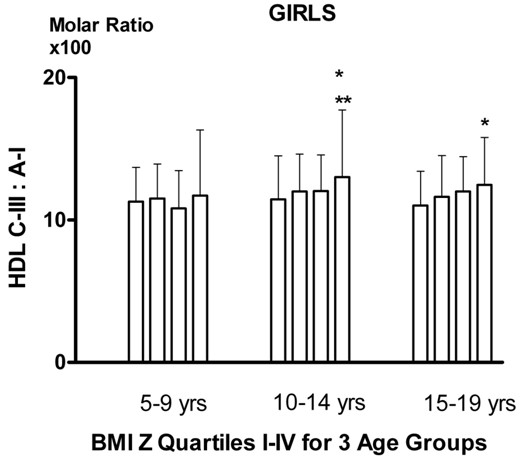
**HDL ApoC-III: ApoA-I molar ratio (x100) by BMI Z quartiles in girls.** * p for trend < 0.05, ** p < 0.05 for difference between quartiles I-III and IV.

ApoA-I was lower in those within the 4^th^ quartile for BMI Z *vs* 1^st^ to 3rd for girls aged 15 to 19 years (p < 0.05) and for boys aged 5–9 and 10–14 years (p < 0.01, Table 2). HDL apoC-III was lower for boys less than 15 years of age (p < 0.05) but higher for 15–19 year-old boys (p < 0.01). HDL apoC-III relative to apoA-I represented by the molar ratio (Figures 1 and 2) showed an increase in girls aged 10–14 years (p < 0.05 for linear trend, p < 0.05 for comparison of those with the 4^th^ quartile for BMI Z *vs* 1^st^ to 3^rd^), which continued at ages 15–19 years (P < 0.05 for trend). In boys the increase occurred at ages 15–19 years (p < 0.01 for trend and for quartile comparison). Log non HDL apoC-III was higher (p < 0.01) in those with BMI Z in the 4^th^ quartile for any sex-age groups, but the ratio of HDL apoC-III to LDL+VLDL apoC-III (C-III ratio) was lower in the 4^th^ quartile (p < 0.05) for any sex-age groups. Multivariate analyses showed that BMI Z was negatively, while total cholesterol and triglyceride positively associated with HDL apoC-III after adjusting for age and sex. The effects of fasting insulin and glucose on the association of HDL apoC-III with BMI Z score were assessed (data not shown). The results showed the association was not affected by fasting glucose for any age groups in both genders. However, the association was reduced by fasting insulin in boys but not girls.

**Table 2:** Apolipoproteins by sex, age group and BMI Z quartiles I-III and IV

	**Sex**	**Age**	**BMI Z quartiles**				
		**(years)**	**I-III**	**n**	**IV**	**n**	**p**
ApoA-I (mg/dl)	Females	5-9	118.3 + 14.1	95	114.3 + 17.7	32	0.2364
		10-14	111.8 + 14.4	138	107.6 + 12.8	46	0.0640
		15-19	116.3 + 20.1	120	109.6 + 17.5	41	0.0465
	Males	5-9	123.5 + 16.1	91	115.6 + 12.2	31	0.0053
		10-14	116.7 + 15.1	135	109.9 + 12.0	45	0.0029
		15-19	110.3 + 15.4	97	106.9 + 15.2	32	0.2720
HDL ApoC-III (mg/dl)	Females	5-9	4.07 + 0.97	95	4.05 + 1.28	32	0.9277
		10-14	4.08 + 0.98	138	4.33 + 1.66	46	0.3282
		15-19	4.17 + 1.23	120	4.19 + 1.27	41	0.9282
	Males	5-9	4.42 + 1.01	91	3.77 + 0.89	31	0.0014
		10-14	4.10 + 1.01	135	3.74 + 0.83	45	0.0181
		15-19	4.02 + 1.05	97	4.63 + 0.99	32	0.0036
Log Non HDL ApoC-III	Females	5-9	0.62 + 0.50	95	0.91 + 0.53	32	0.0081
		10-14	0.82 + 0.47	138	1.16 + 0.63	46	0.0012
		15-19	0.75 + 0.44	120	1.05 + 0.43	41	0.0003
	Males	5-9	0.56 + 0.34	91	0.94 + 0.54	31	0.0010
		10-14	0.72 + 0.44	135	1.05 + 0.57	45	0.0008
		15-19	0.92 + 0.47	97	1.27 + 0.51	32	0.0009
CIII Ratio**	Females	5-9	2.42 + 1.11	95	1.81 + 1.05	32	0.0059
		10-14	1.94 + 0.89	138	1.55 + 1.04	46	0.0207
		15-19	2.03 + 0.76	120	1.56 + 0.72	41	0.0007
	Males	5-9	2.66 + 1.04*	91	1.64 + 0.84	31	0.0001
		10-14	2.13 + 0.90+	135	1.45 + 0.75	45	0.0001
		15-19	1.76 + 0.86#	97	1.39 + 0.64	32	0.0114

** The ratio of HDL apoC-III to VLDL + LDL apoC-III.

The p values are derived from testing the difference in means of those with BMI Z quartiles I-III vs. IV.

## Discussion

Obesity-related increases in HDL apoC-III relative to apoA-I occur earlier in girls from ages 10 to 14 years, followed by boys at ages 15 to 19 years. Since HDL apoC-III is a stronger risk factor for DM2 in women [6], the sex differences are consistent with observations that girls are less protected from DM2 than boys [4]. It is possible that the earlier increase in HDL apoC-III in girls is associated with their known earlier pubertal onset and increased adiposity associated with more insulin resistance at earlier ages than boys [12]. Sex hormone differences are also a possible explanation, but our previous analyses have only documented changes in HDL-C, apoA-I and apoA-II. In boys, total testosterone and free testosterone are associated with decreased HDL-C and apoA-1 during puberty [13,14], concurring with our observations in the same Cherokee population that obesity-related decreases in HDL-C and in lipoproteins containing either apoA-I or both apoA-I and apoA-II were greater in boys aged 15 to 19 years than in girls [15].

Since insulin-resistant conditions such as obesity and DM2 are associated with low HDL-C and generation of smaller HDL particles [16-18], the relative increase in HDL apoC-III may change the HDL particle’s protective functions. However, it is unknown whether the apoC-III content of HDL influences efflux function, but this could be answered by use of a macrophage HDL efflux assay which has shown cholesterol efflux to be superior and independent of HDL-C as a predictor of atherosclerosis [19].

Epidemiological observations supporting a role for LDL uptake and HDL-mediated efflux in the pathogenesis of DM2 have been supported by in vitro studies showing that addition of LDL to isolated human and rat islets decreases glucose stimulated insulin secretion and is attributed to cholesterol uptake by LDL receptors on the β-cell [20]. Furthermore, the effect of intracellular accumulation of cholesterol is influenced by HDL-mediated cholesterol efflux via the adenosine triphosphate binding cassette transporter A1 (ABCA1). Mice lacking the LDL receptor and the ABCA1 transporter were not protected from effects of added LDL on decreasing beta cell insulin secretion, suggesting that HDL-mediated efflux plays a critical protective role [21]. Further studies have revealed that increased cholesterol content in the beta cell membrane down-regulates insulin secretion by influencing membrane depolarization, the signal for calcium influx and calcium-mediated insulin secretion [22]. These studies provide a plausible explanation for the role of HDL in protecting the beta cell from cholesterol-induced toxicity, supporting the hypothesis that compositional changes in HDL consisting of decreased cholesterol but increased apoC-III relative to apoA-I, could result in adverse functional changes. Although we have previously reported cross-sectional observations of non-HDL apoC-III in relation to insulin resistance in Cherokee Indian adolescents [23], HDL apoC-III has not been prospectively evaluated as a risk factor for type 2 diabetes in any population other than Turkish adults in whom it was strongly predictive with a greater effect in women [6]. In the current cross-sectional analysis we could not investigate the predictive effect of HDL apoC-III on outcomes such as the onset of type 2 diabetes. Although the association of HDL apoC-III with BMI Z score was not affected by fasting glucose, the association was affected by fasting insulin in boys but not girls.This may reflect an association with insulin resistance characterized by increased insulin levels before progressing to lower insulin levels with the onset of diabetes, and is consistent with an effect of insulin resistance on apoC-III transcription via failed phosphorylation of the transcription factor, foxo-1 resulting in continued apoC-III transcription [24], thus increasing total and HDL apoC-III. Further analysis showed that obesity related increase in HDL apoC-III was reduced by the effect of insulin in boys suggesting that insulin excess may reduce the effect in boys offering them protection.

Increases in HDL apoC-III may result from increased transfer from surplus non-HDL apoC-III, a particle which we have previously shown to be associated with insulin resistance in children [23] and is known to be a predictor of atherosclerotic lesion progression [25,26]. The apolipoprotein changes with increasing BMI occurred in association with an increased triglyceride and low HDL-C, the classic derangements in lipid transport observed in insulin resistant states. Besides preceding DM2 and occurring in association with the metabolic syndrome and cardiovascular risk [27,28], the criteria were independent risk factors for DM2 in the PROCAM study, and low HDL-C was found to be interactive with obesity in predicting diabetes [5]. Conversely high levels of HDL-C were protective against DM2 in Arizona Pima women but not men [29].

It is also possible that effects of low or abnormal HDL on risk for DM2 are compounded by the effects of an increased BMI, since obesity is a known risk factor and has a quantitative effect on diabetes prediction in women [30] and men [31]. Furthermore onset of obesity before age 21 years compounds diabetes risk [30] and plays a role in causing insulin resistance mediated in part by increased free fatty acids and their deposition in liver and muscle resulting in resistance to insulin’s action on glucose lowering [32]. Evidence from clinical studies supports the role of a sustained elevation in fatty acids resulting in beta cell failure and progression to diabetes [33].

## Conclusions

Our observations provide evidence for a predominantly female obesity-related increase in HDL apoC-III relative to apoA-I, potentially leading to dysfunctional effects on the β-cell and association with increased risk for DM2. The observation of an earlier obesity-related change in girls than in boys is consistent with increased risk for DM2 in females. These changes add to our previously observed obesity-related decreases in HDL-C and apoA-I in both sexes [15] that also may increase risk for diabetes. The data support the hypothesis that when the apoA-I and cholesterol content of HDL are lowered in the obese state, the increase in HDL apoC-III may compound dysfunction and predispose to DM2. The data may provide rationale for prospective cohort studies to establish whether HDL apoC-III and associated change in HDL function predict DM2 and whether lifestyle and pharmacological interventions can improve the abnormalities leading to diabetes prevention in youth and reduction in the increased female prevalence.

## Abbreviations

DM2: Type 2 diabetes; BMI: Body mass index; Z: Z score (computed by formula as a fraction of the standard deviation from the median); HDL: High density lipoprotein; HDL-C: HDL-cholesterol; Apo: Apolipoprotein; LDL-C: Low density lipoprotein cholesterol; Vs: Versus; ABCA1: Adenosine triphosphate binding cassette transporter A1; PROCAM: Prospective Cardiovascular Műnster Study.

## Competing interests

The authors declare that they have no competing interests.

## Author’s contributions

PRB was a co-investigator and was involved in the study design, data collection, the data analysis and manuscript preparation. SK represented the Cherokee Nation and provided support for personnel selection, subject recruitment, data collection and liaison during the study. WW was involved in data interpretation and statistical analysis. PA conducted the lipid and apolipolipoprotein assays and was involved in data interpretation. ETL was the principal investigator for the original Cherokee Diabetes Study and was recipient of the NIH grant to study risk factors in the Cherokee. All authors read and approved the final manuscript.

## Author’s information

PRB is a Professor of Pediatrics in the Section of Diabetes and Endocrinology at the University of Oklahoma Health Sciences Center, A Fellow of the National Lipid Association, Board Certified in Pediatric Endocrinology and Clinical Lipidology and a Member of the Harold Hamm Diabetes Center.

SK is Chief Epidemiologist at the Cherokee Nation

WW is Professor of Epidemiology and Biostatistics at the University of Oklahoma Health Sciences Center

PA is Head of the Lipid and Lipoprotein Laboratories at the Oklahoma Medical Research Foundation.

ETL is Head of the Center for Native American Research at the University of Oklahoma Health Sciences Center and was Principal Investigator for the study.
